# Haemophilus influenzae Urethritis: A Localization to Consider

**DOI:** 10.7759/cureus.42601

**Published:** 2023-07-28

**Authors:** Yassine Ben Lahlou, Zakaria Malihy, Mostapha Benaissa, Mariama Chadli, Mostapha Elouennass

**Affiliations:** 1 Bacteriology, Mohammed V Military Training Hospital, Rabat, MAR

**Keywords:** non-gonococcal urethritis, haemophilus influenzae, oral sex, urethritis, haemophilus influenzae urethritis

## Abstract

Urethritis may be acute or subacute and can be classified as gonococcal or nongonococcal. Nongonococcal urethritis is mainly dominated by *Chlamydia trachomatis*. Other bacteria such as *Haemophilus influenzae* have been implicated in some cases.

We report the case of a 56-year-old diabetic man with *H. influenzae* urethritis following unprotected oral sex. The patient initially received treatment with ciprofloxacin, but this proved to be ineffective. Once our diagnosis was made, we switched the patient to cefixime for a period of seven days, which resulted in a successful clinical outcome.

## Introduction

In cases of nongonococcal urethritis, *Chlamydia trachomatis* is the most prevalent pathogen, followed by *Mycoplasma genitalium*. The *Haemophilus* genus, particularly *Haemophilus influenzae*, has been implicated as a pathogen in acute urethritis. For example, a Japanese study reported a prevalence of 5.2% of *H. influenzae* in cases of acute urethritis [1]. However, it should be noted that the cause of urethritis is not always identified, with up to 35% to 50% of cases remaining without an etiology [2,3].

## Case presentation

Herein, we present the case of a 56-year-old married man with diabetes who sought medical attention due to urethral discharge, urethral discomfort, and dysuria. These symptoms appeared a week after engaging in protected sexual intercourse but unprotected orogenital intercourse.

Upon clinical examination, the patient was apyretic and had no palpable adenopathy or genital ulcers. Urethral discharge, which was clear and abundant, was collected for standard testing and molecular biology assays.

A cytobacteriological examination of the urethral discharge was carried out, which revealed the presence of neutrophils associated with Gram-negative coccobacilli on Gram staining. Additionally, molecular biology testing did not reveal either *C. trachomatis* or *Neisseria gonorrhoeae*.

Inoculation was performed on chocolate agar (Polyvitex®, bioMérieux, Durham, NC, USA) and blood agar. After 24 hours, smooth colorless colonies with entire margins and oxidase-positive and catalase-positive were observed. Species identification was achieved using the Api NH® gallery (bioMérieux) with a 99% confidence level. Furthermore, a cytobacteriological examination of the urine was performed, which was sterile with a nonsignificant leukocyturia of 3,000/mL.

An antibiogram was performed by the diffusion method on chocolate agar Polyvitex® according to the recommendations of the antibiogram committee of the French Microbiology Society CA-SFM 2022. The strain was sensitive to penicillin G, ampicillin, piperacillin-tazobactam, imipenem, ertapenem, and third-generation cephalosporins (cefotaxime, cefepime, and cefixime).

The minimum inhibitory concentrations of ceftriaxone and ciprofloxacin used in the E-test method were 0.016 and 32 mg/L, respectively. The patient was initially treated with ciprofloxacin for a week; however, there were no signs of recovery. After our diagnosis, the patient was treated with cefixime for seven days and exhibited positive clinical progress.

## Discussion

*H. influenzae* is a commensal of the oropharynx and nasopharynx and is a strictly human bacterium. It has a high colonization rate of approximately 80% and is known to cause various infections such as pneumonia, cellulitis, septic arthritis, and septicemia [4,5]. The bacterium has been described for many years as one of the rare agents responsible for nongonococcal urethritis [6].

Transmission through sexual contact has been reported for *H. influenzae* [7], including unprotected oral sex [3,7,8,9]. Our patient had protected coitus but unprotected orogenital sex, which suggests transmission of the oropharyngeal commensal flora from his partner, who was likely colonized by *H. influenzae*, to the urethra, allowing the bacteria to express their virulence factors, mainly adhesins and pili, responsible for adhesion to the genital epithelium. Furthermore, *H. influenzae* produces other pathogenicity factors, especially a protease that cleaves IgA1 from the mucosa, capable of inducing the formation of a biofilm, and whose antigenic variability contributes to immune evasion [4]. Regarding host receptivity factors, we suggest that the patient’s age and the occurrence of the risk factor diabetes contributed to the pathophysiology of the infection.

Clinically, common symptoms of *Haemophilus* spp. urethritis include urethral discomfort, dysuria, and/or mucopurulent or clear discharge [7]. Our patient’s symptoms were consistent with this clinical presentation, exhibiting all of the previously mentioned clinical elements as well as a clear discharge.

Given this bacterium’s fastidious and exigent nature, as with any bacteria that can cause acute urethritis, it is crucial to quickly inoculate the sample on an enriched medium. For our sample, seeding was done on blood agar and chocolate agar Polyvitex®. Moreover, the required factors V and X, also known as nicotinamide adenine dinucleotide and hemin, respectively, were used to confirm the identification of *H. influenzae*.

Beta-lactams, fluoroquinolones, tetracyclines, and aminoglycosides are usually effective antibiotics against the various species belonging to the *Haemophilus *genus and have a good therapeutic value in their infections [4]. Beta-lactamase production is higher in samples taken from the urethra compared to those from the respiratory tract [10]. The emergence of fluoroquinolone-resistant strains makes an antibiogram necessary in most clinical situations, as was the case with our patient, who was initially treated with ciprofloxacin for a week without clinical amelioration. This result led to the investigation of the minimum inhibitory concentration of ciprofloxacin to determine its effectiveness, which confirmed its resistance. The patient’s treatment was switched to cefixime for seven days, resulting in positive clinical improvement

## Conclusions

*H. influenzae* is a rare but frequently overlooked pathogen that may be transmitted through orogenital intercourse, particularly among vulnerable individuals. Culturing on enriched media and antibiotic susceptibility testing are recommended to detect *H. influenzae* infection.

## References

[1] Ito S, Hatazaki K, Shimuta K (2017). Haemophilus influenzae isolated from men with acute urethritis: its pathogenic roles, responses to antimicrobial chemotherapies, and antimicrobial susceptibilities. Sex Transm Dis.

[2] Gerhardt P (2016). Male urethritis. Ann Dermatol Venereol.

[3] French Society of Microbiology (SFM) (2018). Urogenital and Sexually Transmitted Infections. REMIC.

[4] Denis F, Ploy M-C, Martin C, Cattoir V (2016). Haemophilus. Medical Bacteriology. Standard Techniques (3rd edn.).

[5] CMIT CMIT (2020). Haemophilus Influenzae Infections. ECN Pilly, 27th edn.

[6] Ducours M, Puges M, Desclaux A, Barthod L, Peuchant O, Cazanave C (2020). Haemophilus spp., an emerging multidrug-resistant sexually transmitted pathogen. Med Mal Infect.

[7] Vives A, da Silva GV, Alonso-Tarrés C, Suarez JB, Palmisano F, Cosentino M (2021). Haemophilus urethritis in males: a series of 30 cases. Rev Int Androl.

[8] Bradshaw CS, Tabrizi SN, Read TR, Garland SM, Hopkins CA, Moss LM, Fairley CK (2006). Etiologies of nongonococcal urethritis: bacteria, viruses, and the association with orogenital exposure. J Infect Dis.

[9] Deza G, Martin-Ezquerra G, Gómez J, Villar-García J, Supervia A, Pujol RM (2016). Isolation of Haemophilus influenzae and Haemophilus parainfluenzae in urethral exudates from men with acute urethritis: a descriptive study of 52 cases. Sex Transm Infect.

[10] Deguchi T, Ito S, Hatazaki K (2017). Antimicrobial susceptibility of Haemophilus influenzae strains isolated from the urethra of men with acute urethritis and/or epididymitis. J Infect Chemother.

